# Choroidal Biomarkers: A Repeatability and Topographical Comparison of Choroidal Thickness and Choroidal Vascularity Index in Healthy Eyes

**DOI:** 10.1167/tvst.9.11.8

**Published:** 2020-10-08

**Authors:** Katharina Breher, Louise Terry, Thomas Bower, Siegfried Wahl

**Affiliations:** 1 Institute for Ophthalmic Research, University of Tübingen, Tübingen, Germany; 2 School of Optometry and Vision Sciences, Cardiff University, Cardiff, UK; 3 School of Engineering, Cardiff University, Cardiff, UK; 4 Carl Zeiss Vision International GmbH, Aalen, Germany

**Keywords:** choroidal thickness, choroidal vascularity index, OCT

## Abstract

**Purpose:**

Choroidal thickness (ChT) and choroidal vascularity index (CVI) represent two important metrics in health-, disease-, and myopia-related studies. Wide-field swept-source optical coherence tomography (OCT) provides improved and extended imaging and extraction of choroidal variables. This study characterizes the topography and repeatability of these parameters in healthy eyes.

**Methods:**

Swept-source OCT volume scans were obtained on 14 young adult patients on three separate days. ChT and CVI were automatically corrected for image magnification and extracted for different enface regions within an extended ETDRS grid of 10 mm diameter. Topographical distribution, correlation to ocular length, and intersession repeatability of both choroidal parameters were assessed.

**Results:**

CVI showed little fluctuation between subfields, unlike ChT, which demonstrated thinning toward the peripheral choroid (coefficients of variation 5.92 vs. 0.89). ChT showed a consistent negative correlation with axial length (ρ = −0.05 to −0.61), although this was only statistically significant in the inner superior subfield (*P* = 0.02). There was no consistent or significant relationship between CVI and axial length or between CVI and ChT. The repeatability of CVI measurements (3.90%–5.51%) was more consistent between scan regions than ChT measurements (10.37–20.33 µm).

**Conclusions:**

CVI values were consistent across the central 10 mm of the retina, while ChT reduced with eccentricity. The repeatability of both parameters is similar to the effect size reported in many studies using the choroid as a biomarker, which should be considered in the interpretation of findings.

**Translational Relevance:**

This study provided normative as well as metrological information for the clinical interpretation of ChT and CVI in health and disease.

## Introduction

The choroid is located between the retina and the sclera and consists of five layers—Bruch's membrane, choriocapillaris, Haller's layer, Sattler's layer, and the suprachoroid—with its main task to provide blood supply to the outer retina.^1^^,^^2^ The choroid is suggested to play an essential role in various fields of human eye research. Changes to the choroidal structure have been suggested as biomarkers for a variety of ocular pathologies; altered choroidal thickness (ChT) was previously observed in central serous chorioretinopathy,^3^ Vogt-Koyanagi-Harada disease,^4^ and polypoidal choroidal vasculopathy and exudative age-related macular degeneration.^5^ Moreover, bidirectional changes of ChT influence the regulation of eye growth due to its location in the signaling pathway from retina to sclera.^2^^,^^6^

However, the exact physiologic mechanisms behind the observed ChT changes still require further clarification. It is assumed that the fast alterations are based on adjustments of the vascular rather than stromal components.^2^^,^^7^^–^^10^ Therefore, choroidal vascularity developed as a further subject of interest in human eye research. The term *choroidal vascularity index* (CVI) was introduced as the ratio of luminal area to total choroidal area. The higher the CVI, the more vascular tissue compared with stromal tissue is present in the choroid.^11^ CVI was previously investigated in a healthy study population,^12^ as well as in ocular pathologies, such as diabetic retinopathy,^13^ central serous chorioretinopathy,^14^ age-related macula degeneration,^15^ panuveitis,^16^ and retinitis pigmentosa.^17^ While there are only a few studies existent, they report only vague and somewhat inconsistent association between choroidal vascularity and myopia.^18^^–^^21^

ChT and CVI can be obtained from optical coherence tomography (OCT) images. After segmentation of the choroidal area, the hyporeflective blood vessel lumina are distinguished from the hyperreflective stromal tissue. The reliability of the results from graphical analysis strongly depends on the OCT technology (scan depth, scan resolution, and image contrast)^11^^,^^22^ but also on subject factors (absolute choroidal thickness and the pigmentation of the retinal pigment epithelium).^23^^,^^24^ Therefore, these parameters influence the repeatability of ChT and CVI measurements. The repeatability metric helps to differentiate between measurement noise and true measured effect, which is pertinent for interpreting study findings. This becomes essential when effect sizes are small, in combination with high intersubject variability, as is typical of studies of the choroid.

Therefore, the purpose of this study is to evaluate the repeatability of ChT and CVI measurements metrologically and to put them into the context of commonly found effect sizes in choroidal research. Moreover, the topographic distribution across an extended scan area, as well as the relationship between choroidal parameters and axial length, is assessed. To our knowledge, this is the first study for this purpose using automated analysis of widefield volume scans from swept-source OCT.

## Materials and Methods

### Study Participants

The prospective and cross-sectional study adhered to the Declaration of Helsinki and was approved by the ethics committee of the Faculty of Medicine of the University of Tübingen. Written informed consent was obtained from all participants prior to data collection. Fifteen patients without known ocular pathologies were enrolled in the study. One patient was excluded from analysis due to erroneous choroidal segmentation resulting from small pupil size and thus reduced image quality. The study participants included in the analysis had a mean age of 27 ± 3 years (range, 24–37 years) and an average axial eye length (AEL) of 24.83 ± 0.91 mm (range, 23.12–26.52 mm).

### OCT Scanning Protocol

Participants underwent nine OCT volume scans on their undilated right eye, using swept-source OCT (ZEISS PlexElite 9000; Carl Zeiss Meditec, Inc., Dublin, CA, USA). The OCT device uses a central wavelength between 1040 and 1060 nm and a sweep range from 980 to 1120 nm, resulting in an increased scan depth of 3 mm. The axial and lateral resolutions in tissue are 6.3 µm and 20 µm, respectively. Three consecutive scans were performed, at the same time of day on three separate days, to control for diurnal variations in choroidal structure.^25^^,^^26^ Moreover, participants were advised to avert caffeine^27^^,^^28^ and nicotine^29^ at least 1 hour prior to the measurement to avoid short-term changes of ChT. In order to wash out near work-induced accommodative choroidal thinning^30^^,^^31^ and to stabilize blood pressure,^26^ participants completed a 10-minute resting phase with distance viewing. For the current study, a 12 × 12-mm isometric volume scan centered on the fovea was chosen. This scan pattern consisted of 1024 B-scans with 1024 A-scans per B-scan. Eye tracking, enhanced-depth imaging, and follow-up mode were enabled to reduce motion artifacts, to enhance the visibility of the choroid, and to ensure a constant scanning area throughout all scans. The same experienced examiner obtained all scans. Furthermore, participants moved their head off of and back onto the chinrest and headrest between each scan.

### Extraction of Choroidal Thickness

Choroidal segmentation was performed automatically by image processing of each volume scan via the Advanced Retinal Imaging Network (ARI Network; Carl Zeiss Meditec, Inc.). The ARI Network represents a research portal provided by Carl Zeiss Meditec, Inc., which offers various algorithms for researchers using the PlexElite 9000 OCT device. The choroidal segmentation algorithm uses a graph-based approach with a combination of intensity, axial gradient, gradient magnitude, and positional information to segment the retinal pigment epithelium (RPE) and the choroidal-scleral interface. To save time and increase segmentation reliability in volume scans with multiple adjacent B-scans, a subset of B-scans is segmented first. The remaining B-scans are segmented considering the results from the first subset. Exemplary central and peripheral B-scans of the scan were checked for segmentation errors by the examiner.^32^

**Figure 1.**
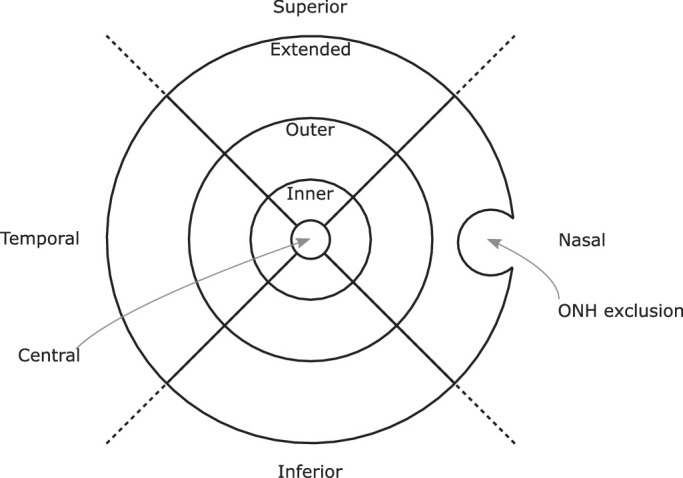
Extended ETDRS grid, showing the optic nerve head exclusion zone for a right eye. The ring diameters are 1 mm, 3 mm, 6 mm, and 10 mm.

The choroidal thickness map for the entire scan area was extracted as a two-dimensional matrix in pixels and was further processed in MATLAB (MATLAB 2020a; The MathWorks, Inc., Natick, MA, USA). An axial conversion factor of 1.9531 from pixel to microns was applied, which is based upon the ratio of true scan depth (3000 µm) to digital image scan depth (1536 pixels). Subsequently, a modified extended Early Treatment of Diabetic Retinopathy Study (ETDRS) grid^33^ was overlaid on the segmented choroidal thickness map (Fig. 1). It consisted of four rings with baseline radii of 0.5 mm (central), 1.5 mm (inner), 3.0 mm (outer), and 5.0 mm (extended). The three outer rings were further divided into nasal, temporal, superior, and inferior sectors. The ETDRS grid size was adjusted for the AEL (equation 2) of the participant (ZEISS IOLMaster 700; Carl Zeiss Meditec AG, Jena, Germany) to compensate for the individual ocular magnification. Data from the optic nerve head (ONH) was selected and subsequently excluded via a semiautomated approach due to inherent segmentation errors in this region. The ONH was excluded by presenting the examiner with an en-face stack image and prompting the user to select with their cursor two points that define the diameter of a circle. This circle was later used to define an exclusion zone for calculations of CVI. These steps are described in more detail in the following section.

### Extraction of Choroidal Vascularity Index

The novel CVI calculation algorithm is written in MATLAB (MATLAB 2019b; The MathWorks, Inc.) and makes use of the Parallel Computing Toolbox for parallelized “for” loops with slices of the volume sent to each worker node in turn for processing. The user interface for this code is in the form of Windows dialogues and figure plots, which request cursor click positions for various tasks. The code also relies on the preparation of a data file, listing the filenames of the volume scans, along with the AEL of each scan. The scan itself is stored as an 8-bit unsigned grayscale image. The procedure for calculating CVI for an image once it is loaded into MATLAB is adapted from the protocol of Sonoda et al.^34^^,^^35^ and is outlined below.

The volume scan is extracted from the input file and stored in a three-dimensional matrix. The RPE segmentation and choroidal thickness map are stored in a matrix, and the segmentation for the choroidal-scleral boundary is calculated by summing the RPE segmentation matrix and the choroidal thickness matrix. The AEL is also extracted from the input files. To give the algorithm a baseline to quantify the grayscale value of a typical fluid, the average voxel value is taken in the two non-en-face planes of the scan, at 50% of the width of the slice, and 75% of the height of the slice (assumed to be within the vitreous). This value is referred to as the “liquid norm.” A mask is then created that specifies the region of interest (ROI) for analysis. From the RPE and choroidal segmentation data, a three-dimensional binary mask matrix is generated, holding a value of 1 within the ROI and 0 outside the ROI. For normalization of the image, the liquid norm is used as a lower threshold on the volume scan (i.e., all values below the threshold were set to zero), and voxel values were rescaled between 0 and 255. To reduce speckle and assist with thresholding, a median blur was applied to each slice in the scan using a 3 × 3 window. The volume scan was binarized using Niblack auto local thresholding^36^ over each slice of the scan, whereby a moving circular window of radius 20 pixels specified the binarization threshold. The threshold, *T*, was calculated from the local mean, *m*, and local standard deviation, *σ*, of the pixels in the window, along with a parameter *k*, which was set to 0.2.

(1) T=m+k×σ.

Any value above the threshold was set to 1, and values below were set to 0, thus producing a binarized image. This study made use of an extended ETDRS grid, centered on the fovea, with an extended diameter of 10 mm and exclusion of the optic nerve head region (Fig. 1).

Careful lateral scaling was performed for each image, to account for the effects of ocular biometry on OCT magnification.^37^ In the lateral dimension, the length each pixel, represented in mm *p_mm_*, was given by

(2) $$p_{mm}=\;\frac{\left(l_{a}-\;\Delta_{cp}\right)\;\theta_{air}}{d_{p}n_{B}},$$

where *l_a_* is the AEL, *Δ_cp_* is the distance between the cornea and principal plane and is given as 1.82 mm,^38^ θ*_air_* is the scan angle (in radians) of the OCT in air and is 40° for this study, *d_p_* is the dimension of the en-face scan in pixels, and *n_B_* is the bulk refractive index of the ocular humors, which is taken as 1.336. The 13 ETDRS grid regions were stored as two-dimensional Boolean mask matrices. Each matrix was also masked against the optic nerve head exclusion circle; typically, this intersected with the extended nasal grid cell. The CVI was calculated from each voxel column of an en-face view to create a map of CVI for the scan. Within the ROI of each column, the number of voxels representing the vessel lumen was counted, *n_v_*, and the total number of voxels in the ROI column was counted, *n_ROI_*. The CVI for each column was calculated as

(3) $$CVI_{col}=\;\frac{n_{v}}{n_{RoI}}\;\times 100.$$

This CVI map was stored and presented as an image with an ETDRS grid overlay. Finally, the CVI for each of the ETDRS regions was calculated by summing the total number of voxels representing the vessel lumen in the grid region within the ROI, *N_v_*, and the total number of voxels in the ROI within each region, *N_ROI_*. The CVI for each region was calculated as

(4) $$CVI_{reg}=\;\frac{N_{v}}{N_{RoI}}\;\times 100.$$

### Statistical Data Analysis

MATLAB (MATLAB 2020a; The MathWorks, Inc.) was used for statistical analysis. Normality of the data was tested with the Lilliefors test. For the analysis of topographical distribution, measurements from all nine scans were averaged per patient and are presented as a median and interquartile range. The quartile-based coefficient of variation^39^ (CV) was calculated (equation 5) where *Q_25_* and *Q_75_* denote the 25th and 75th quantiles of the distribution.

(5) $$CV=\left(\frac{Q_{75}-\;Q_{25}}{Q_{75}+\;Q_{25}}\right)\times 100.$$

   Correlations of the choroidal parameters with AEL were performed using Spearman rank correlation.^40^ The coefficient of repeatability (CR) from nonnormal data was derived for each ETDRS region as explained elsewhere.^41^^,^^42^ In short, the median of each of the three measurements per day and its difference to the overall mean across the 3 days were calculated. Subsequently, the resulting deltas were evaluated in a cumulative distribution function. The 95% limits of the cumulative distribution function were determined and multiplied by $\sqrt{3/2}$ as a correction factor for centered data. The CR was considered half the length of the 95% interval. It is noteworthy that only intersession but not intrasession repeatability was considered in the analysis, to reflect most studies that investigate choroidal metrics in an intersession rather than intrasession (consecutive) manner.

## Results

### Widefield Topography of ChT and CVI

Presented ChT and CVI were sorted by retinal location and eccentricity, as presented in Table 1. In general, the ChT decreased with increasing eccentricity and was typically highest superiorly, followed by the temporal, inferior, and nasal areas. In contrast, CVI showed a relatively uniform pattern with almost no eccentricity- or region-dependent fluctuations. Quartile-based CV across the scanned retina were 5.92 for ChT and 0.89 for CVI. There were no significant correlations found between ChT and CVI in any ETDRS sector (all *P* > 0.05). However, there appears to be a general transition from negative to increasingly positive correlations between the parameters with increasing eccentricity.

**Table 1.** Topographical Distribution of ChT and CVI across the Scan Area and Their Correlation with Each Other

	ChT (µm)		CVI (%)		Correlation ChT × CVI	
Characteristic	Median	IQR	Median	IQR	ρ	*P* Value
Central field						
Central	323.24	29.30	72.89	4.51	−0.39	0.17
Inner fields						
Superior	333.98	54.69	74.20	4.12	−0.06	0.84
Inferior	299.80	64.45	74.12	4.58	−0.38	0.18
Temporal	315.43	48.83	75.11	3.75	−0.14	0.64
Nasal	298.82	54.69	73.78	4.61	−0.24	0.41
Outer fields						
Superior	329.10	64.45	73.80	4.49	+0.30	0.30
Inferior	303.71	111.33	74.15	4.14	−0.17	0.57
Temporal	297.85	50.78	76.05	5.65	+0.12	0.69
Nasal	246.09	78.12	76.08	5.35	−0.07	0.82
Extended fields						
Superior	344.72	70.31	73.35	3.72	+0.25	0.38
Inferior	285.15	64.45	73.49	3.95	+0.28	0.33
Temporal	287.11	46.87	75.34	6.01	+0.46	0.10
Nasal	200.19	60.55	74.72	5.44	+0.38	0.19

IQR, interquartile range.

**Figure 2.**
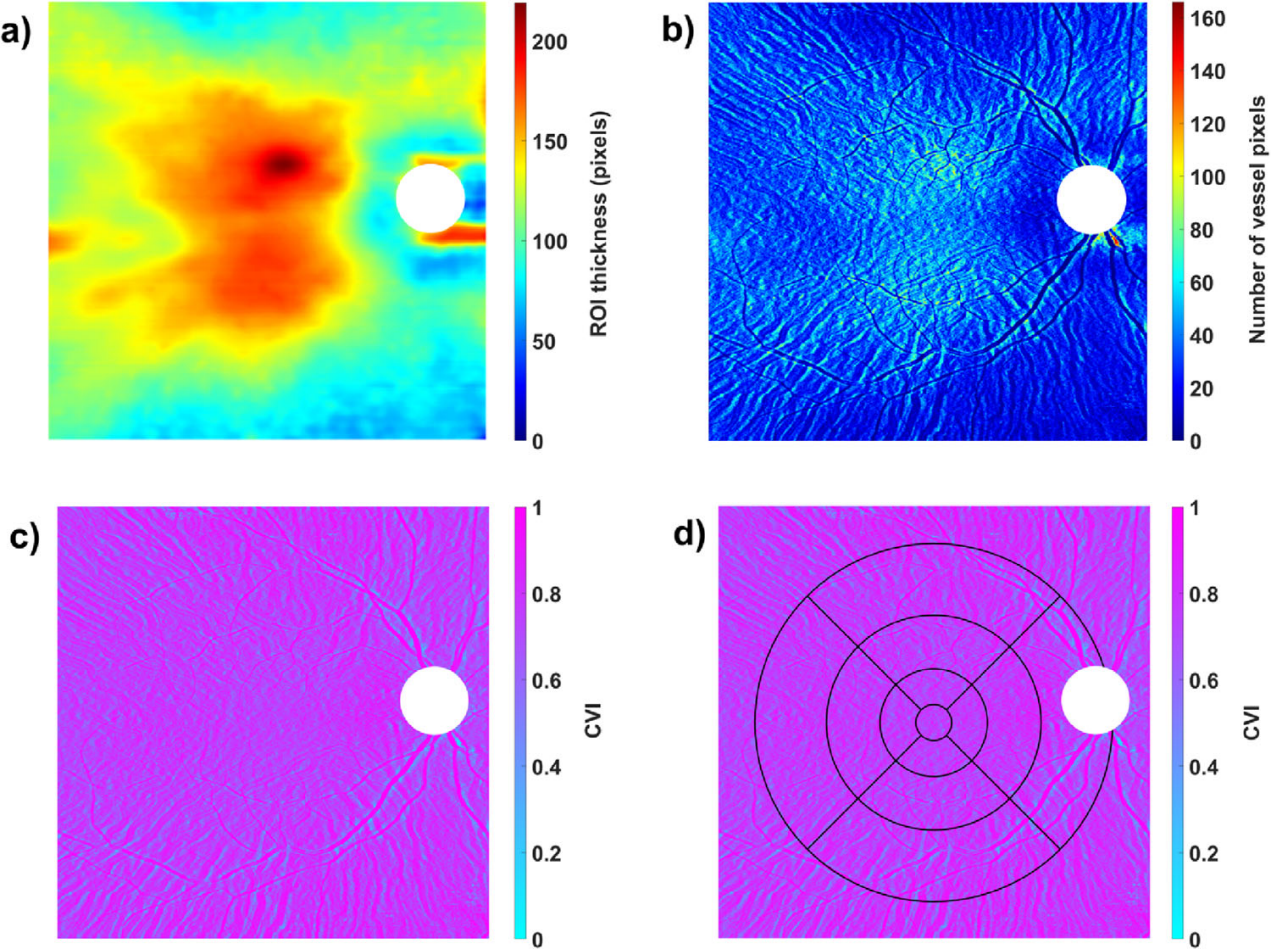
Output of the CVI algorithm for an example participant: (a) ChT segmentation as ROI. (b) Calculated number of vessel pixels. (c) CVI map without ETDRS grid. (d) CVI map with overlaying ETDRS grid, adjusted for image magnification. The white area of the ONH was excluded from analysis.

Figure 2 shows an example of the CVI algorithm output with typical topographic variations for ChT and CVI. As depicted in Figure 2b and Figure 2c, the structures of larger choroidal vessels are highly visible with a subsequently higher CVI. This relationship is not surprising, since these pixel columns will comprise predominantly vessel lumina, yielding a high CVI in these localized areas.

### Relationship between Axial Length and Choroidal Metrics

The relationship of both choroidal parameters with AEL was investigated to explore the potential effects of eye size on ChT and CVI. While ChT decreased with increasing AEL in all scan fields, CVI showed a more variable relationship with AEL (Table 2). The central and inner fields correlated positively with AEL, whereas negative correlations were found in the most peripheral regions. Despite the consistency in the pattern of ChT, the relationship with AEL was statistically significant only in the inner superior field (*P* = 0.02), and there were no significant correlations between CVI and AEL.

**Table 2.** Spearman Correlation Coefficient (ρ) and Associated *P* Value of Choroidal Metrics with Axial Eye Length

	ChT × AEL		CVI × AEL	
Characteristic	ρ	*P* Value	ρ	*P* Value
Central field				
Central	−0.49	0.08	+0.45	0.10
Inner fields				
Superior	−*0.61*	*0.02*	+0.16	0.57
Inferior	−0.25	0.38	+0.34	0.24
Temporal	−0.48	0.08	+0.21	0.47
Nasal	−0.45	0.11	+0.46	0.10
Outer fields				
Superior	−0.37	0.19	−0.24	0.42
Inferior	−0.21	0.47	+0.24	0.42
Temporal	−0.40	0.15	−0.05	0.86
Nasal	−0.47	0.09	+0.22	0.44
Extended fields				
Superior	−0.13	0.65	−0.34	0.24
Inferior	−0.05	0.87	−0.06	0.83
Temporal	−0.32	0.26	−0.27	0.35
Nasal	−0.50	0.07	−0.14	0.63

Significant correlations are highlighted in italics.

### Intersession Repeatability

The CR between the three measurement sessions for choroidal metrics is reported in Table 3. There were small regional fluctuations for ChT, ranging from 10.37 µm (inner temporal and extended inferior fields) to 20.33 µm (extended nasal field). CVI repeatability ranged between 3.90% in the outer inferior field and 5.51% in the temporal extended field. The quartile-based CVs for the repeatability across the total scan area were 11.11 and 9.66 for ChT and CVI, respectively. The last two columns of Table 3 represent the relative proportion between the repeatability coefficient and the absolute median values from Table 1. With exception of the nasal extended subfield, ChT exhibits a relatively better repeatability than CVI.

**Table 3.** CR for ChT and CVI across the ETDRS Sectors

	CR		CR/Median	
Characteristic	ChT (µm)	CVI (%)	ChT (%)	CVI (%)
Central field				
Central	11.96	4.00	3.70	5.49
Inner fields				
Superior	16.35	4.20	4.89	5.65
Inferior	13.95	4.21	4.65	5.67
Temporal	10.37	4.54	3.29	6.05
Nasal	10.76	4.15	3.60	5.62
Outer fields				
Superior	13.95	3.93	4.24	5.33
Inferior	12.76	3.90	4.20	5.26
Temporal	11.36	5.41	3.81	7.11
Nasal	11.56	4.88	4.70	6.41
Extended fields				
Superior	11.16	5.00	3.24	6.81
Inferior	10.37	4.39	3.64	5.97
Temporal	11.16	5.51	3.89	7.32
Nasal	20.33	4.02	10.16	5.38

The last two columns denote the proportion in percentage between the CR and the median value reported in Table 1.

## Discussion

The study undertaken investigated the widefield distribution of ChT and CVI, their intersession repeatability, and their relationship to axial eye length. Moreover, novel and advantageous methodology was applied as follows:

(1) Swept-source OCT with an increased widefield scan area of 12 × 12 mm was used. A smaller region of interest with a diameter of 10 mm was evaluated due to potential information loss resulting from magnification correction using AEL. To our knowledge, this is the largest evaluated retinal area for CVI distribution, ChT and CVI repeatability, and axial length scaling correlation to date.
(2) The analysis of three-dimensional volume scans was possible thanks to enhanced scan depth and thus improved image quality compared to spectral-domain OCT. Previously, most CVI and ChT metrics were obtained from line scans or as single measurement points only.
(3) All algorithms used were optimized to run entirely automated (with the exception of the semiautomated exclusion of the optic nerve head) on all 1024 B-scans per volume. This includes image-processing steps for choroidal thickness and choroidal vascularity analysis, as well as the subsequent scaling of the ETDRS grid to account for image magnification. This approach reduces analysis time, examiner bias, and interobserver variation.
(4) The size of the ETDRS sectors was compensated for image magnification for each participant, from their AEL measurement. Moreover, the study measurements were controlled for choroidal diurnal rhythm by performing the measurements always at the same time of day for each participant. Eye movement artifacts during and between scans were minimized by using the inbuilt eye-tracking and follow-up mode of the device.

### CVI Reveals Less Topographical Variations Than ChT

ChT showed a higher variability of topographical distribution across the retina compared to CVI. ChT generally decreased toward the periphery (particularly in the nasal sector), as reported previously.^43^^–^^46^ CVI distribution patterns were more uniform with no obvious influence of retinal location and thus lower variability metrics. Regional consistency is also reflected in the low quartile-based CV of 0.89 for CVI, compared to 5.92 for ChT. This is consistent with earlier studies, although these found generally lower absolute CVI values of around 45% to 65%.^12^^,^^18^^,^^47^^,^^48^ These differences likely arise from the use of different OCT devices, scan patterns, image-processing algorithms, and participant age and ethnicity distributions. However, it has been stated that central CVI (measured in two dimensions) is not significantly influenced by the choice of scan pattern^49^ or OCT device.^50^ Moreover, ChT and CVI showed a tendency of eccentricity-dependent association with each other, with a shift from negative to positive correlations toward the peripheral retina. This suggests that the thicker central and inner choroid results from a higher stromal rather than vascular component. This is in contrast to previously reported positive correlations of ChT and CVI in the central retina up to 1.5 mm diameter,^12^ which is comparable to the central and inner fields defined in the current study.

### Eccentricity-Dependent Correlations of AEL with CVI

When establishing and adjusting normative databases, the interactions of choroidal metrics with AEL are an important factor to be considered. Like this, it is possible to differentiate physiologic from truly pathologic deviations. ChT is known to thin with increasing AEL.^12^^,^^51^^–^^53^ The lack of statistical significance in the current study, except in the inner superior field, most likely arises from the relatively small sample size. CVI again exhibits mixed associations with AEL; centrally, longer ocular length leads to an increased vascular component and/or reduced stromal component. In contrast, higher AEL comes along with lower CVI in the peripheral retina. Interestingly, this eccentricity-dependent correlation pattern is the inverse of the ChT and CVI correlation described above. Previous correlation findings appear somewhat inconclusive; low negative correlations of CVI with AEL have been reported,^12^^,^^18^^,^^47^ while slightly increased vascular components with myopia have also been observed.^20^^,^^21^ However, these findings are all limited to the central or maximally the outer scan fields and do not all reach statistical significance.

### Repeatability of Choroidal Metrics Is Similar to Reported Effect Sizes

The analysis of intersession repeatability was included for two reasons: first, to evaluate whether there are more metrologically preferred retinal areas for measurements in future studies. These are areas that exhibit lower CR values. Second, the repeatability of these choroidal metrics is put into context of commonly found effect sizes. To address the first question, the nasal extended area manifests the highest CR with 20.33 µm compared to the other ETDRS sectors (10.37–16.35 µm). One earlier publication reported an average CR of 30 µm for ChT with automated segmentation in swept-source volume scans but without differentiating between ETDRS areas.^54^ Moreover, another methodologically similar study found the opposite for spectral-domain OCT scans, with the best repeatability in the nasal sectors due to thinner choroids and thus advantageous imaging properties.^42^ However, comparisons to other past literature are limited, since the range of methodologies used and analyses reported are broad. In contrast to ChT, repeatability of CVI measurements did not show any regional variations, surprisingly not even in the extended nasal subfield. Therefore, CVI can be measured irrespective of the retinal region from a metrologic perspective. There are few published reports on the repeatability of CVI measurements and all limited to spectral-domain and line scan analysis, making comparisons to the present study difficult. Moreover, these commonly evaluate the repeatability of luminal and stromal areas separately, which causes difficulties when translating it to repeatability results of final and summarized CVI measurements. However, the somewhat most comparable study found 95% limits of agreements ranging from approximately −4% to +3%.^49^ From a relative perspective, however, ChT exhibits a better repeatability compared to the absolute thickness than CVI (Table 3). This finding is in line with another study in healthy participants.^12^ There has not been a definite theory for this relation yet. However, one hypothesis suggests that the repeatability of CVI is dependent on the repeatability of ChT as a first and inherent processing step. Therefore, this finding can be expected. The second step was to compare reported effect sizes with measurement variability. Only if the CR does not exceed the effect size can measurement noise be neglected as a source of error. Typical reported differences between healthy and disease conditions are around 2% to 6% for CVI (review: Agrawal et al.^11^). For ChT, the effect size is highly dependent on the condition studied. In retinal disease, ChT alterations of around 100 µm are commonly found.^3^^–^^5^ However, in myopia research, the measurement reliability is similar to or even exceeds published effect sizes of maximally 20 µm (review: Read et al.^55^), even when using swept-source OCT technology with improved choroidal imaging. In summary, measurement repeatability should be carefully considered when interpreting “true” change or difference in both choroidal parameters and distinguishing from measurement variation.

### Limitations

A relatively small sample size of *n* = 14 was used for this study. Although axial length was normally distributed and covered the range of usual emmetropic and myopic ocular sizes in the normal population, the small sample size could affect the homogeneity of the sample. It is therefore prudent to view the correlation results as indicative rather than definitive, and these relationships warrant further investigation with a larger sample with a wider range of axial lengths and stronger homogeneity. All included participants were young adults without ocular pathology and from Caucasian descent (except for one Indian participant). Therefore, the findings should be applied cautiously to other age groups and ethnicities, as well as in pathology. Moreover, participants could not be fully controlled for all factors shown to affect the choroid, such as smoking and caffeine. By enrollment into the study, participants were advised to avoid caffeine and nicotine intake at least for 1 hour before the measurements, but this could not be verified by the examiner. Blood pressure as another confounding factor was not measured specifically, as it was assumed to be stable within one individual because of the 10-minute resting and distance viewing period prior to the measurements. Despite imaging advances with swept-source and long-wavelength OCT, there were still B-scans in the evaluated subset with limited visibility of either the choroidal-scleral interface or vessels. The limited contrast of choroidal structures occurred due to OCT signal roll-off with increasing scan depth and therefore measurement noise. Retinal vessel shadows (areas of hyporeflectivity in the choroid from overlying structures) represent another measurement artifact and could lead to a misjudgment of CVI. However, these artifacts are limited to the extended nasal, inferior, and superior analysis regions. While this factor might have a small influence on CVI in those regions, it should not affect the repeatability results per se, as the retinal vessels are not transient. Therefore, these artifacts were acknowledged in these three out of 13 analysis regions but were not removed from the images due to their expected limited effect on the outcomes. The results of the extended nasal sector might have been additionally influenced by the semiautomated exclusion of the optic nerve head, leading to a reduced number of analyzed pixels in that region, which varied between participants based on the size of that feature. Despite the image size compensation for magnification, caused by axial length differences of the participants, they were not corrected for ocular curvature. Subsequently, this might lead to small biases in measurements of absolute ChT, especially in the periphery of the scan and with increasing curvature. However, the magnitude of error usually tends to be relatively small with up to 6.0 µm at a scan eccentricity of almost 7 mm.^56^ Moreover, no highly myopic eyes were included into the study and only affects the absolute measures of ChT but not the repeatability measurements, as these were analyzed in a relative manner to each other. Therefore, the total error in the current study is existent but most probably has only very limited effect on the final outcomes and conclusion.

## Conclusion

This study provides evidence that choroidal vascularity does not follow a clear and distinct topographical pattern across the central retina, unlike ChT. Moreover, mixed correlations between ChT and CVI, as well as CVI and axial length, were found, whereas choroidal thinning with increasing axial length was confirmed. It was further observed that the repeatability of CVI measurements does not vary across scan areas, in contrast to ChT measurements. However, the repeatability of both metrics is similar to or even exceeds previously reported effect sizes in many studies using choroidal parameters. Measurement repeatability should be considered in the interpretation of results from studies involving the choroid as a biomarker.
